# Changes in free amino acid and monoamine concentrations in the chick brain associated with feeding behavior

**DOI:** 10.1186/s40064-015-1058-8

**Published:** 2015-06-12

**Authors:** Phuong V Tran, Vishwajit S Chowdhury, Mao Nagasawa, Mitsuhiro Furuse

**Affiliations:** Faculty of Agriculture, Laboratory of Regulation in Metabolism and Behavior, Graduate School of Bioresource and Bioenvironmental Science, Kyushu University, Fukuoka, 812-8581 Japan; Division for Experimental Natural Science, Faculty of Arts and Science, Kyushu University, Fukuoka, 819-0395 Japan

**Keywords:** Food intake, Free amino acid, Monoamine, Brain, Neonatal chick

## Abstract

Domesticated chicks are precocial and therefore have relatively well-developed feeding behavior. The role of hypothalamic neuropeptides in food-intake regulation in chicks has been reported for decades. However, we hypothesized that nutrients and their metabolites in the brain may be involved in food intake in chicks because these animals exhibit a very frequent feeding pattern. Therefore, the purpose of this study was to examine the feeding behavior of chicks as well as the associated changes in free amino acid and monoamine concentrations in the chick brain. The feeding behavior of chicks was recorded continuously for 6 h. The next day, brain and blood samples were collected when the chicks either attempted to have food (hungry group) or turned food down (satiated group), in order to analyze the concentrations of the free amino acids and monoamines. We confirmed that the feeding behavior of neonatal chicks was characterized by short resting periods between very brief times spent on food intake. Several free amino acids in the mesencephalon were significantly lower in the satiated group than in the hungry group, while l-histidine and l-glutamine were significantly higher. Notably, there was no change in the free amino acid concentrations in other brain regions or plasma. As for monoamines, serotonin and norepinephrine were significantly lower in the mesencephalon of the hungry group compared with the satiated group, but 5 hydroxyindolacetic acid (5-HIAA) was higher. In addition, serotonin and norepinephrine levels were significantly higher in the brain stem of the hungry chicks compared with the satiated group, but levels of 5-HIAA and homovanillic acid were lower. Levels of both dopamine and its metabolite, 3,4-dihydroxyphenylacetic acid, were significantly higher in the diencephalon and telencephalon of the chicks in the hungry group. In conclusion, the changes in the free amino acids and monoamines in the brain may have some role in the feeding behavior of neonatal chicks.

## Background

The appetite biology of birds is generally assumed to have particular similarities with that of mammals (Denbow 1984; Furuse 2002). Therefore, understanding appetite regulation in birds may also be helpful for human appetite biology. In addition, it is very important to understand the physiological mechanism of chicks’ feeding behavior because chicks are precocial and therefore they demonstrate well-developed feeding behavior from the very beginning of their life. Chicks feed frequently in the course of a day, with much time spent on both feeding and resting. However, to the best of our knowledge, there are no data available regarding this interesting phenomenon.

There are many reports concerned with the role of hypothalamic neuropeptides in food-intake regulation (Bungo et al. 2000, 2004; Furuse et al. 1997a, b, 1999, 2000; Kawakami et al. 2000a, b; Kuenzel et al. 1987; Tachibana et al. 2008, 2009). While it could be predicted that a certain period of time is needed for the changes of neuropeptides in the brain to occur, it has been reported that 120 min was needed for significant increments to occur of the gonadotropin-inhibitory hormone (GnIH) mRNA, a food-intake-stimulating neuropeptide (Tachibana et al. 2005; McConn et al. 2014), in the quail diencephalon in vitro (Chowdhury et al. 2010). Therefore, it is difficult to explain the frequent food intake and resting behavior observed in chicks in terms of the action of neuropeptides; rather, quick changes of metabolites in the brain may contribute to this process. In the central nervous system (CNS), several amino acids are known to be important neurotransmitters. Potential roles of amino acids in neuronal regulation have recently received much attention. For instance, the exogenous administration of l-arginine, l-ornithine, l-alanine, l-serine, l-cysteine and glycine has proved that these amino acids have sedative and hypnotic effects (Asechi et al. 2006; Kurauchi et al. 2006; Suenaga et al. 2008a, b). On the other hand, several amino acids serve as excitatory neurotransmitters; these include l-glutamate and l-aspartate, that induce neuronal activity through their stimulation (Monaghan et al. 1989). It is also possible that the influence of sedation or excitation may be involved in feeding behavior. In addition, some amino acids have been shown to influence food intake in neonatal chicks. Central injection of l-proline increased food intake in an ad libitum feeding condition and decreased food intake under a fasting condition (Haraguchi et al. 2007). Central administration of l-tryptophan reduced food intake (Bungo et al. 2008), but l-leucine stimulated food intake (Izumi et al. 2004). Furthermore, some metabolites of amino acids, such as monoaminergic products or gaseous nitric oxide, were also reported to be involved in the central control of feeding behavior (Choi et al. 1994; Leibowitz and Alexander 1998; Meguid et al. 2000; Wellman 2000). Therefore, we hypothesized that quick metabolic changes, such as changes in amino acid and monoamine concentrations in the brain, may be associated with the frequent feeding and resting behavior of neonatal chicks. To test this hypothesis, in the present study, we first aimed to clarify feeding behavior of neonatal chicks by measuring the time spent on feeding and resting and further examined the changes in free amino acid and monoamine concentrations in different brain regions that are associated with chicks’ feeding behavior.

## Methods

### Animals and food

Day-old male layer chicks (Julia strain; *Gallus gallus domesticus*) were purchased from a local hatchery (Murata Hatchery, Fukuoka, Japan). The birds were housed in groups in metal cages at a constant temperature of 30 ± 1°C under continuous light until they were 2 days old. Food (Commercial starter diet [metabolizable energy: 12.77 MJ/kg and protein: 24%; food ingredients: grain 61% (mainly maize), defatted meal 25% (soybean meal and maize gluten meal), fish meal 9%, rice bran 1% and others 4%; AX, Toyohashi Feed and Mills Co. Ltd., Aichi, Japan]) and water were provided ad libitum. When they were 3 days old, the chicks were placed in individual cages to acclimatize for 2 days before the experiment was started. This study was performed in accordance with the guidance for Animal Experiments in the Faculty of Agriculture of Kyushu University, and Law No. 105 and Notification No. 6 of the Japanese government.

### Experimental procedure

The experiment was conducted for 2 days with a total of sixteen chicks. On the first day of the experiment, the feeding behavior of chicks (5 days old) was recorded continuously for a total of 6 h. Chicks were provided with food and water ad libitum during the experimental time. Video cameras were positioned vertically on the top of the experimental units to record the feeding behavior of chicks on a digital versatile disc without causing any disturbance to the chicks. On the second day of the experiment, the same chicks (6 day old) were first randomly distributed into two groups based on their body weight so that the average body weight between the groups was as uniform as possible. One group was labeled the hungry group, and members of this group were immediately removed from the cage once they attempted to have food. The other group was labeled the satiated group, and members of this group were removed from the cage as soon as they turned down food. Once chicks were removed from the cage after either attempting to have food or turning down food, they were immediately killed by exposure to isoflurane (Mylan Inc., Tokyo, Japan). Blood was immediately collected from the jugular vein into heparinized tubes and centrifuged at 4°C and 10,000*g* for 4 min. Then the plasma was collected and stored at −80°C until it was analyzed to determine the concentrations of free amino acids and monoamines. The brains were dissected, and the telencephalon, diencephalon, mesencephalon, cerebellum and brain stem were collected and stored in Eppendorf tubes. The five brain regions were identified as described elsewhere (Chowdhury et al. 2014; Kuenzel and Masson 1988). Then these brain samples were frozen in liquid nitrogen and stored at −80°C in a deep freezer until their analysis for free amino acid and monoamine concentrations.

### Amino acid analysis

Amino acids in the plasma and brain samples were determined according to the method described by Ohmori et al. (2011). Brain samples were weighed and then homogenized in 0.2 M perchloric acid. The homogenates were placed on ice for 30 min and then centrifuged at 4°C and 20,000*g* for 15 min. The brain supernatant was collected and filtrated through 0.2 µm filters (Millipore, Bedford, USA). A 20-µl portion of filtrate from each brain sample was dissolved with 2 µl of 1 M NaOH and then vortexed in order to mix thoroughly for the amino acid analysis. Plasma samples were centrifuged with ultrafiltration tubes (Amicon Ultra-0.5, Millipore, Bedford, USA) to collect the supernatants for analysis. Free amino acid concentration was measured using a UPLC (the Acquity™ UPLC system, comprised of Waters Binary Solvent Manager, Water Sample Manager and Waters FLR Detector) with an ACCQ-TAG™ ULTRA C18 1.7 µm 2.1 × 100 nm column (Waters Corporation, USA). The excitation and emission wavelengths used for fluorescent detection of amino acids were 350 and 450 nm, respectively. The system operated with a flow rate of 0.25 ml/min at 30°C. The UPLC gradient system (A = 50 µm sodium acetate (pH 5.9), B = methanol) was 10–20% B over 3.2 min, 20% B for 1.2 min, 20–40% B over 3.6 min, 40% B for 1.2 min, 40–60% B over 3.8 min, 60% B for 1 min and 60–10% B over 0.01 min. Before the UPLC analysis, each 10 µl of brain and plasma sample was transferred to a UPLC tube, to which 20 µl *N*-acetylcysteine/*O*-phthalaldehyde and 70 µl borate buffer were added, and then it was left for 2 min in a dark room. Standard solutions were treated by the same method. The amino acid concentrations were expressed in nmol/ml for plasma samples, and in pmol/mg wet tissue for brain samples.

### Monoamine analysis

Monoamines such as dopamine (DA) and its metabolites, 3,4-dihydroxyphenylacetic acid (DOPAC), homovanillic acid (HVA), norepinephrine (NE), and the NE metabolite 3-methoxy-4-hydroxyphenylglycol (MHPG), as well as serotonin (5-HT) and its metabolite 5 hydroxyindoleacetic acid (5-HIAA), were determined in the brain regions as described by Tomonaga et al. (2008). The supernatants that were collected and separated during amino acid analysis were used for monoamine measurement. The supernatants were adjusted to approximately pH 3 by the addition of 1 M sodium acetate and then filtrated through 0.2-µm filters (Millipore, Bedford, USA). 30 µl of filtrate from each brain sample were applied to a high performance liquid chromatography (HPLC) system (Eicom, Kyoto, Japan) with a 150 × 2.1 mm octadecylsilane (ODS) column (EICOMPAK SC-5ODS; Eicom) and an electrochemical detector (ECD-300, Eicom, Kyoto, Japan) at an applied potential of +0.75 V versus an Ag/AgCl reference analytical electrode. The changes in electric current (nA) were recorded on a computer using an interface system (Power Chromver 2.3.2.J: AD Instruments, Tokyo, Japan). The mobile phase (pH 3.5), containing 0.1 M sodium acetate, 0.1 M citric acid, 100 mg/ml sodium 1-octane sulfonate, 5 mg/ml disodium ethylenediaminetetraacetic acid and methanol, was used at a flow rate of 0.2 ml/min. The external standard solution, consisting of 100 pg/ml each of DA, DOPAC, NE, MHPG, HVA, 5-HT and 5-HIAA, was determined with reference to the retention time and height of the peaks of the tissue homogenates. The detection limit of the system for all monoamines was 0.1 pg/sample. The monoamine concentrations in the brain samples were expressed in pg/mg wet tissue.

### Statistical analysis

All data were statistically analyzed by two-way ANOVA. A t test was used for the analysis when a significant interaction was detected. Experimental data in each group were first subjected to a Thompson rejection test to eliminate outliers (*p* < 0.01), and the remaining data were used for the analysis. Statistical analysis was conducted using a commercially available package, StatView (version 5, SAS Institute, Cary, USA 1998). Significance was set at *p* < 0.05. Data were expressed as mean ± SEM.

## Results

### Feeding behavior of neonatal chicks

**Figure 1:**
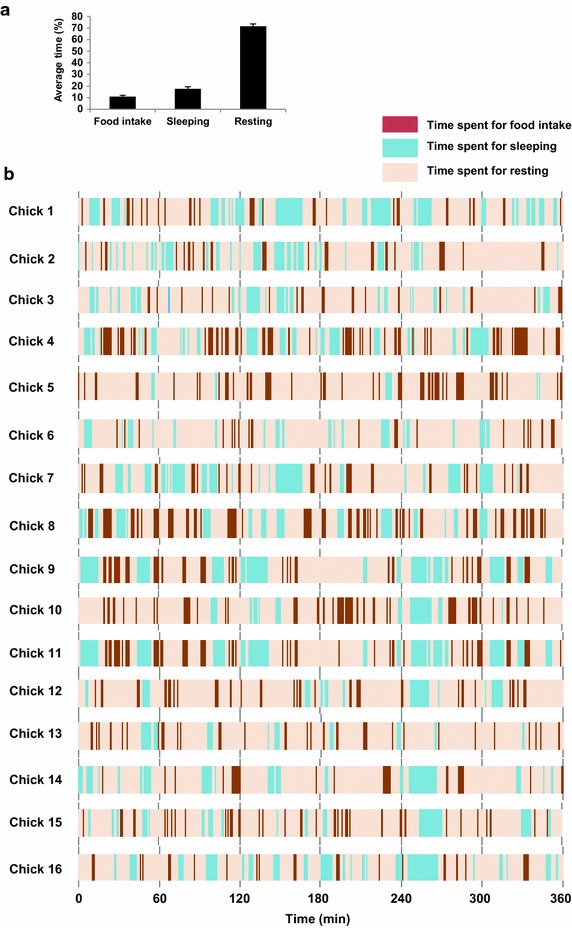
The feeding behavior of neonatal chicks observed at 6 h. The average time spent for food intake, sleeping and resting (**a**) and the precise feeding behavior of sixteen individual chicks (**b**) are shown.

The average time spent for food intake, sleeping and resting occupied 10.9, 17.5 and 71.5%, respectively during the 6 h of the experimental period is shown in Figure 1a. The precise feeding behavior of neonatal chicks during 6 h of observation is shown in Figure 1b. It was confirmed that the feeding behavior of neonatal chicks was characterized by frequent food intake, with short resting and sleeping periods in between. It was observed that the maximum time spent on food intake was about 4 min (Figure 1b). On the other hand, the minimum time spent on food intake was 4 s, although it is not possible to see this very short time in Figure 1b as it is a compressed version of the 6 h of data. The maximum time spent on resting and sleeping in between food intake was about 36 min and the minimum amount of time was about 18 s.

### Free amino acid concentrations in the brain

**Figure 2:**
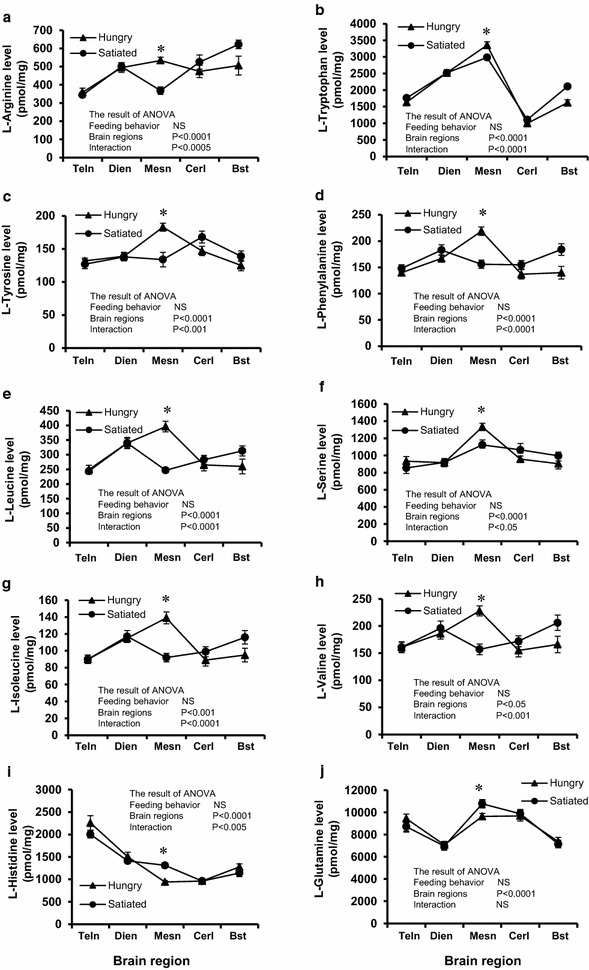
Changes in the free amino acid concentrations in the chick brain corresponding to feeding behavior. Levels of l-arginine (**a**), l-tryptophan (**b**), l-tyrosine (**c**), l-phenylalanine (**d**), l-leucine (**e**), l-serine (**f**), l-isoleucine (**g**), l-valine (**h**), l-histidine (**i**), l-glutamine (**j**) were determined in different brain regions. Data are expressed as mean ± SEM of 7–8 chicks per group. *Teln* telencephalon, *Dien* diencephalon, *Mesn* mesencephalon, *Cerl* cerebellum, *Bst* brain stem, *FB* feeding behavior, *BR* brain region, *NS* not significant. *p < 0.05.

The changes in the free l-amino acid concentrations in different brain regions during hungry and satiated states are shown in Figure 2 and Table 1. It was found that the concentrations of some l-amino acids significantly differed between hungry and satiated groups only in the mesencephalon. l-Arginine, l-tryptophan, l-tyrosine, l-phenylalanine, l-leucine, l-serine, l-isoleucine and l-valine were significantly higher in the hungry group than in the satiated group. These amino acids also showed significant interactions between feeding behavior and brain region, which indicates that hunger influenced the mesencephalic increment of these amino acids (*p* < 0.01) (Figure 2a–h). In contrast, mesencephalic l-histidine and l-glutamine showed significantly lower concentrations in the hungry group (*p* < 0.05) (Figure 2i, j). Mesencephalic l-histidine also showed a significant interaction, suggesting that the lower level of this amino acid in the mesencephalon was caused by the state of the appetite. Table 1 shows the changes in levels of amino acids that showed significant differences between brain regions. However, l-alanine and GABA were significantly higher in the mesencephalon of hungry chicks (*p* < 0.0001). There was no significant difference between the hungry and satiated groups in terms of the concentrations of free amino acids in the plasma.

**Table 1:** Changes in free amino acid concentrations (pmol/mg wet tissue) in the brain regions corresponding to feeding behavior in chicks

Amino acids	Telencephalon		Diencephalon		Mesencephalon		Cerebellum		Brain stem		*P* value		
	Hungry	Satiated	Hungry	Satiated	Hungry	Satiated	Hungry	Satiated	Hungry	Satiated	FB	BR	Interaction
l-Aspartic acid	3,379 ± 189	3,217 ± 113	2,769 ± 136	2,811 ± 279	3,897 ± 83	3,952 ± 102	2,721 ± 136	2,856 ± 87	3,030 ± 204	3,196 ± 174	NS	<0.0001	NS
Taurine	9,051 ± 526	8,606 ± 365	3,713 ± 175	3,697 ± 112	2,810 ± 41	3,062 ± 138	7,016 ± 286	7,174 ± 167	3,402 ± 204	3,566 ± 203	NS	<0.0001	NS
l-Alanine	856 ± 58	787 ± 28	686 ± 31	675 ± 35	716 ± 22	566 ± 22	766 ± 51	787 ± 37	676 ± 52	749 ± 29	NS	<0.0001	NS
GABA	3,303 ± 196	3,190 ± 68	5,128 ± 161	5,052 ± 221	6,027 ± 154	5,354 ± 135	2,061 ± 102	2,194 ± 95	3,237 ± 264	3,492 ± 189	NS	<0.0001	NS

Values are mean ± SEM. n = 7–8 in each group.

*FB* feeding behavior, *BR* brain region, *NS* not significant.

### Monoamine concentrations in the brain

Table 2 shows the changes in monoamines and their metabolites in different brain regions corresponding to the feeding behavior. In the mesencephalon, the concentration of 5-HT, NE was significantly higher in the satiated group of chicks, while the concentration of 5-HIAA was lower (*p* < 0.01). However, in the brain stem, the concentrations of 5-HIAA and HVA were significantly higher in the satiated group (*p* < 0.01), and the concentrations of NE and 5-HT were significantly lower (*p* < 0.05). Feeding behavior significantly increased DA and DOPAC in the diencephalon of hungry chicks (*p* < 0.05). However, HVA was significantly higher in the diencephalon and brain stem of those in the satiated group than in those in the hungry group (*p* < 0.01). There were significant interactions between feeding behavior and brain regions (*p* < 0.01) in terms of the concentrations of HVA, NE, 5-HT and 5-HIAA, indicating that alteration of these monoamines is influenced by the state of the appetite and the brain region involved.

**Table 2:** Changes in monoamine concentrations (pg/mg wet tissue) in the brain regions corresponding to feeding behavior in chicks

Monoamine	Diencephalon		Telencephalon		Mesencephalon		Cerebellum		Brain stem		*P* value		
	Hungry	Satiated	Hungry	Satiated	Hungry	Satiated	Hungry	Satiated	Hungry	Satiated	FB	BR	Interaction
DA	161 ± 4	140 ± 9	385 ± 4	339 ± 14	–	–	–	–	147 ± 18	146 ± 11	<0.05	<0.0001	NS
DOPAC	25 ± 3	18 ± 2	16 ± 3	14 ± 1	–	–	–	–	–	–	<0.05	<0.005	NS
HVA	119 ± 7	160 ± 10	89 ± 7	102 ± 5	34 ± 2	23 ± 1	17 ± 1	18 ± 1	2,181 ± 34	2,866 ± 56	<0.0001	<0.0001	<0.0001
NE	–	–	–	–	117 ± 3	199 ± 13	–	–	482 ± 42	322 ± 8	NS	<0.0001	<0.0001
5-HT	405 ± 13	380 ± 26	455 ± 11	436 ± 13	122 ± 11	207 ± 8	43 ± 1	39 ± 1	432 ± 36	338 ± 23	NS	<0.0001	<0.0005
5-HIAA	308 ± 27	321 ± 15	106 ± 5	130 ± 6	248 ± 8	156 ± 7	68 ± 2	63 ± 1	233 ± 24	350 ± 17	NS	<0.0001	<0.0001

Values are mean ± SEM. n = 7–8 in each group.

*DA* dopamine, *DOPAC* 3,4-dihydroxyphenylacetic, *HVA* homovanillic acid, *NE* norepinephrine, *5-HT* serotonin, *5-HIAA* 5-hydroxyindoleacetic acid, *FB* feeding behavior, *BR* brain region, *NS* not significant.

## Discussion

It is generally known that food intake in birds is a frequent process. However, there are no reports showing the precise characteristics of this phenomenon. In the current research, it was found that the feeding behavior of neonatal chicks is particularly characterized by short resting intervals between very brief periods of food intake. Domesticated chicks are considered to be a precocial nestling type among the avian species, with the ability to search for their own food immediately after hatching (Furuse 2002). There is ample research on appetite showing that the CNS plays a vital role in the regulation of food intake and energy expenditure (Furuse 2002; Harrold et al. 2012). However, a certain period of time may be needed for the regulation of neuropeptides (Chowdhury et al. 2010). Thus, it seems inappropriate to explain the frequent feeding behavior of neonatal chicks in terms of neuropeptide signaling in the CNS or peripheral nervous system activating the neural circuitry or endocrine system that regulates food intake and energy homeostasis. In the present study, we therefore focused on revealing the metabolic changes in amino acids and monoamines in the brain that enable the quick regulation of food intake in neonatal chicks.

The concentration of many amino acids was significantly higher in the mesencephalon of those chicks in the hungry group. The administration of exogenous amino acids to the brain stimulates feeding behavior in chicks (Haraguchi et al. 2007) and thus there is a possibility that an increment in endogenous amino acid may have some influence on food intake in hungry chicks. It is well known that the hypothalamus in the diencephalon possesses numerous appetite-associated neuropeptides and their target receptors (Chowdhury et al. 2012; Meister 2007; Parker and Bloom 2012). In addition to the hypothalamus, there are several other brain sites that have been proven to have roles in feeding behaviors, such as the brain stem and the sensory circumventricular organs. The brain stem contains the so-called satiety region that transfers signals from the gastrointestinal tract via the vagus nerve to produce a sense of satiety (Richards and Proszkowiec-Weglarz 2007). Furthermore, GnIH, which is an orexigenic neuropeptide in birds (McConn et al. 2014), showed a strong immunoreactive fiber distribution throughout the diencephalon and mesencephalon in the quail brain (Ukena et al. 2003). Therefore, not only diencephalon but also the mesencephalon would have an important role in food-intake regulation. In particular, the changes in free amino acids in the mesencephalon in both the hungry and the satiated states in the current study indicate that the mesencephalon may be a novel site that also contributes to the regulation of food intake in neonatal chicks.

In the present study, it was observed that the concentration of several amino acids was lower in the mesencephalon in the satiety state in comparison with the hungry state. The low amino acid level in the satiety state has the following three possible explanations: (1) the free amino acid pool may be utilized for protein synthesis; (2) some amino acids may be quickly metabolized after feeding and the metabolites may have some sedative and hypnotic effects which induced sleep or anorexigenic effects; (3) the reduction of these amino acids may be related to the increase in other amino acids caused by competition at transporter levels. The declining l-arginine concentration in the mesencephalon in the satiated state was possibly associated with its quick metabolism. l-Arginine can be catabolized to l-ornithine, l-citrulline, l-proline, nitric oxide (NO), l-glutamate and so on (Morris 2004). However, birds lack carbamyl phosphate synthetase, one of the enzymes of the urea cycle necessary for the synthesis of l-citrulline from l-ornithine in the liver and kidney (Tamir and Ratner 1963). Therefore, birds cannot synthesize l-arginine, but can synthesize l-ornithine from l-arginine (Suenaga et al. 2008b). Intracerebroventricular (i.c.v.) injection with l-arginine proportionally increased l-ornithine concentrations in chick brains 10 min post injection, and l-ornithine has sedative and hypnotic effects (Suenaga et al. 2008b). Thus, it could be inferred that under the satiety state, l-arginine was converted to l-ornithine to induce sleepiness that inhibited feeding. On the other hand, l-arginine is also metabolized to l-citrulline and produces NO, which is an important gaseous neurotransmitter (Bredt et al. 1990). NO stimulates the CNS to increase the capacity for food intake (Choi et al. 1994).

l-Tryptophan is an essential amino acid in animals, including birds, and it is known to have two main metabolic pathways: one is the serotonin pathway for the production of 5-HT and its metabolites, and the other is the kynurenine pathway. In the present study, l-tryptophan was lower in the mesencephalon of the satiated group, but 5-HT was higher. Brain 5-HT release has a positive correlation with the availability of brain l-tryptophan (Schaechter and Wurtman 1990). Central administration of l-tryptophan suppressed food intake, with the involvement of 5-HT (Bungo et al. 2008). We therefore assume that l-tryptophan was metabolized to 5-HT in the mesencephalon when the chicks felt satiated and left the feeders. l-Tyrosine is known to be metabolized to l-phenylalanine in the liver. In this study, it was found that the level of l-tyrosine was low in the mesencephalon of the satiated group, while its metabolite NE was higher in the same group. Thus, it could be predicted that l-tyrosine was metabolized to NE and epinephrine under the satiety state. l-Tyrosine was found to increase in both the telencephalon and the diencephalon after 3 h of fasting in a study where it was suggested that fasting was involved in the inhibition of brain tyrosine hydroxylase (Hamasu et al. 2009). Thus, it could be assumed that hunger might have influenced the higher l-tyrosine content in the mesencephalon. Among the altered amino acids, l-histidine and l-glutamine had different responses. Significantly lower concentrations of these two amino acids were observed in the hungry state. l-Histidine is known to be the precursor of histamine that is believed to be involved in energy balance as a neurotransmitter (Sakata et al. 1994). Moreover, l-histamine is activated by a decrease in glucose (Nishibori et al. 1986). Therefore, it could be predicted that l-histidine is the substrate to the synthesis of l-histamine when the chicks tend to feel hungry and come to the feeders.

The depression of DA has been found to induce anorexigenic effects in neonatal chicks (Bungo et al. 2001). This finding partly agreed with the results of the present study, where DA was significantly lower in the diencephalon and telencephalon in the satiety state compared with the hungry group. The changes to the monoamines in the brain stem that corresponded to the different feeding behaviors were the opposite of those taking place in the mesencephalon. HVA and 5-HIAA were significantly higher in the satiety state in the brain stem. As the brain stem can regulate the satiety response (Richards and Proszkowiec-Weglarz 2007), it could be assumed that the brain-stem HVA or 5-HIAA content may contribute in some way to inducing satiety in chicks.

In conclusion, we determined that the precise feeding behavior of neonatal chicks is characterized by frequent food intake punctuated by intervals of resting and sleeping. The quick metabolic changes that were found to occur in the brain amino acid and monoamine concentrations may contribute to the feeding behavior. In particular, in chicks, the mesencephalon may be an important novel brain site related to the control of feeding behavior through metabolic activity. A future study will aim to clarify the functional importance of the altered free amino acids and monoamines for feeding behavior in neonatal chicks.
